# Prevalence of anxiety, depression, and stress among students of Jahangirnagar University in Bangladesh

**DOI:** 10.1002/hsr2.559

**Published:** 2022-03-14

**Authors:** Md. Moyazzem Hossain, Md. Asraful Alam, Monirul Hasan Masum

**Affiliations:** ^1^ Department of Statistics Jahangirnagar University Savar Dhaka Bangladesh; ^2^ Faculty of Business Administration International Standard University Dhaka Bangladesh

**Keywords:** chi‐square, DASS‐21 scale, mental health problems, structural equation modeling, university students

## Abstract

**Background and Aims:**

Anxiety and depression, as well as stress, are well‐known problems observed across the world, particularly among students. This study intends to identify the level of anxiety, depression, and stress among university students and determine its association with their sociodemographic characteristics.

**Methods:**

The primary data were collected from 351 students with the help of a self‐administrated questionnaire consisting of sociodemographic information and contains the Depression, Anxiety and Stress Scale‐21 Items (DASS‐21) instrument over the period December 8, 2019–January 23, 2020. The *χ* ^2^ test is employed to find the association between the status of stress, anxiety, and depression level with selected sociodemographic variables, and confirmatory factor analysis is used to find interrelationships between DASS items.

**Results:**

The results illustrate that no students have an extremely severe level of stress. However, the majority have a mild or moderate level of stress and it is associated with sex and residence (urban or rural). More than 40% of students have extremely severe anxiety. Results reveal that gender, residence (urban or rural), and family type of the students are linked with the anxiety level. The results also suggest that the type of accommodation of the students, their family type, and birth order are related to their depression level at a 5% level of significance. The findings also disclose that female students have more levels of depression, stress, and anxiety than their counterparts.

**Conclusion:**

Considering the finding, the authors think that the university authority should pay a need for greater interest to the mental well‐being of students to enhance their quality of life. Given the harmful impacts of stress on academic performance and health, university administrators should be incorporating anxiety, stress, and depression management training in orientation activities.

## 1 INTRODUCTION

Psychiatric disorders have been one of the primary causes of disability around the world.^1^ The World Health Organization's recently released mental health action plan for 2013–2020 highlights the need for a concerted attempt to enhance mental health based on evidence.^2^ Psychological health is determined by the level of anxiety, depression, and stress. Anxiety is an uncomfortable state of inner chaos that is often escorted by nervous behavior such as walking back and forwards, physical symptoms, and rumination.^3^ Is not fairly the same as fear, which is a reaction to an actual or perceived immediate danger/threats but also includes the possibility of a future threat.^4^ The first step in managing a person having anxiety symptoms includes assessing the probable existence of an inherent medical reason, whose detection is vital to decide the appropriate treatment.^5^, ^6^ Symptoms of anxiety may conceal a natural disease, or as a consequence of a medical disorder.^5^, ^6^, ^7^, ^8^ Previous research on twins has shown that both individual and specific settings have a significant impact on anxiety, with shared environments operating during childhood, but declining towards adolescence.^9^ Child abuse and neglect, a family history linked to mental health illnesses, and poverty are examples of specific quantifiable “environments” that are linked to anxiety.^10^ Furthermore, anxiety is associated with drug misuse, including caffeine, and alcohol, as well as benzodiazepines, which are commonly prescribed to treat anxiety.^9^, ^11^

Another component of determining the status of mental health is depression and it is one of the most common psychiatric issues in the world.^12^, ^13^ Every year, 350 million people are affected by depression.^14^ Young adults face both depression and stress while they remain in a transition period of identity creation.^15^ They are confronted with a variety of behavioral, emotional, sexual, academic, economic, and social issues.^16^ College and university students, in particular, are more prone to suffer from depression as well as stress^17^, ^18^, ^19^ for the reason that they are coping with the social and academic needs to make a proper plan for their professional careers.^20^ Depression among students is caused by a variety of circumstances, for example, more academic demands, cope up with a new environment, and social life also works as a source of depression among students.^19^ Furthermore, socioeconomic determinants have a direct influence on the frequency of depression among students; for example, research has shown that students belonging to the lower socioeconomic classes have a greater rate of depression due to financial insecurity.^21^ New university students are required to adapt to numerous psychosocial adjustments along with coping with social and academic needs.^20^

Likewise, anxiety and depression, stress is also acting as a risk factor for health and well‐being. In the 1950s, Selye^22^ initially disseminated the concept of stress. Stress is a mental or physical event that is the result of one's engagement with the environment and is caused by one's cognitive assessment of the stimulation.^23^ Stress, on the other hand, according to Chang's Dictionary of Psychology Terms, is a state of physical or mental strain that impacts a person's emotional anguish or even perception of pain.^24^ A study pointed out that stress is a complicated phenomenon that largely differs on one's temperaments, experiences, situations, and environmental circumstances.^25^

Several studies tried to find out the prevalence as well as determinants of stress, anxiety, and depression among university students all over the world. The symptoms of stress and anxiety are more common along with moderate to severe levels than depression among the students in Pakistan.^26^ Herrmann et al.^27^ find out the rate of anxiety, depression, and stress symptoms among students in France. Cheung et al.^28^ mention that among subgroups of university students, community college transfer students had the highest degree of anxiety, stress, and depression in China. Students in Malaysia were found to have moderate to severe levels of anxiety, stress, and depression.^29^ Moreover, the incidence of anxiety, stress, and depression was just above 60% among the students of Fayoum University of Egypt.^30^ Islam et al.^31^ assessed the level of depression as well as anxiety among the students of first‐year in Bangladesh. Moreover, several previous studies on mental health among students of the university have mostly explored that sex,^32^, ^33^ academic,^34^, ^35^, ^36^ accommodation,^37^ and geographical^38^ differences may be acted as a contributing factor for health and well‐being status. Furthermore, depression, stress, and anxiety were found to have strong associations with demographic, health‐related, and lifestyle characteristics.^39^ The mental health disorders were more prevalent among female, rural, low‐income, and academically underperforming students.^40^, ^41^, ^42^ The outbreak of coronavirus disease 2019 (COVID‐19) has impacted people's quality of life and style of living around the world. Students at universities are frequently regarded as the future of society, and their mental health should be handled carefully. Given the significant frequency of anxiety, depression, and stress among students, researchers recommend that it is necessary to keep a close eye on their mental health to avoid disastrous effects.^43^ Previous studies pointed out that a higher level of psychological problems was seen among Bangladeshi students during this pandemic condition.^40^, ^44^

The existing literature depicts that the status of student's mental health is associated with their background characteristics. In Bangladesh, some of the universities have been considering the mental health and well‐being of students; however, most of the university authorities ignore this issue. Moreover, it is necessary to understand the prevalence of mental health issues and distress among Bangladeshi university students, as well as which subgroups are most vulnerable and the nature of student's psychological difficulties for taking proper actions to prevent the psychological difficulties and support them. Therefore, this study intends to determine the prevalence of anxiety, stress, and depression among university students, as well as the relationship between these factors and the student's socioeconomic situation. Furthermore, a confirmatory factor analysis (CFA) was carried out to find the interrelationships between Depression, Anxiety and Stress Scale (DASS) items in accordance with their common underlying factors.

## 2 METHODS

The primary data were accumulated from the students of Jahangirnagar University, Bangladesh utilizing stratified random sampling techniques with different courses in different academic years. The authors applied the following formula to compute the minimal sample size for this study because one of the aims is to determine the prevalence (proportion) of depression, stress, and anxiety among students, $n=\frac{pqz^{2}}{d^{2}}$, where n is the required sample size, z is the standard normal variate (which is 1.96 at 95% confidence interval [CI]), p is the expected proportion of students having anxiety, depression, and stress (there is no previous information that is why this study used 0.5 since it provides the largest sample size), and d the margin of error (which is 5%). Hence, the formula provides the required sample size as 385 and the authors send the questionnaires to the selected students, but among them, there are 34 missing questionnaires, that is, the response rate is more than 90%. So, the final sample size for this study is 351. Before starting the data collection, participants were asked to partake in the study voluntarily. They were made ensured about the confidentiality of their information and individual identity as well. The data collection process began after providing consent to partake in the study. The primary data used in the analysis of this paper was collected by well‐trained five graduate students. First of all, the authors organized a training session regarding data collection procedures and then they conduct a face‐to‐face interview to gather the data from December 8, 2019 to January 23, 2020. The data were collected with the help of a self‐administrated questionnaire comprised of two segments. The first part was based on sociodemographic characteristics, such as sex, current domicile, and place of origin (urban/rural), as well as family living systems and parents' educational backgrounds. The DASS‐21 instrument was utilized in the second section. The 23 statistics is used to check the association between variables considered in this study and 5% level of significance and two‐sided tests are considered for hypothesis testing purposes. Moreover, the *χ* ^2^ and normed *χ* ^2^, root mean square error of approximation (RMSEA), root mean square residual (RMR), normed‐fit index (NFI), comparative fit index (CFI), goodness‐of‐fit index (GFI),  and adjusted goodness‐of‐fit index (AGFI) were used for checking the fitted model. Data analysis is performed using IBM SPSS Statistics for Windows (version 23.0) and IBM SPSS Amos (version 24.0).

The DASS‐21 scale is widely applicable to measures stress, anxiety, and depression levels. In the DASS‐21 there are seven items for depression, anxiety, and stress. The depression levels of respondents are categorized as normal (0–9), mild (10–13), moderate (14–20), severe (21–27), and extremely severe (28 and more); anxiety levels are classified as normal (0–7), mild (8–9), moderate (10–14), severe (15–19), and extremely severe (20 and above); and stress is broken down as normal (0–14), mild (15–18), moderate (19–25), severe (26–33), and extremely severe (34+).^45^

## 3 RESULTS

Reliability analysis was performed to establish the items' overall or even internal reliability as a representation of the instrument's stability and consistency in measuring the concept. Internal reliability was measured using Cronbach's *α* developed by Lee Cronbach in 1951,^46^ which increased in value as the intercorrelation between items became more intense. The values of Cronbach's α for depression, anxiety, stress, and overall are 0.72, 0.83, 0.67, and 0.78, respectively. Table 1 demonstrates the frequency distribution of the socioeconomic characteristics of the respondents. Among the 351 respondents, 61% were male and most of the students were staying at the residential Hall/mess (85.2%). However, approximately half of the students are the second child of their parents. This study considers five faculties of Jahangirnagar University and almost 20% of students were selected from each faculty. The results presented in Table 1 depict that 70% of students did not involve in smoking, approximately 70% of students reported that their health condition was good, and only 4% of the students said that they are sick. More than 60% of the students said that they did not involve any kind of physical exercise regularly. Among the respondents, the majority (85.2%) lived in the residential halls or mess (Table 1).

**Table 1:** Frequency distribution of the socioeconomic characteristics of the respondents

	Frequency	%		Frequency	%
Sex of respondent			Birth order		
Male	214	61.0	First child	122	34.8
Female	137	39.0	Second child	169	48.1
Total	351	100.0	Third child	42	12.0
Academic year			Fourth and higher	18	5.1
First year	77	21.9	Total	351	100.0
Second year	82	23.4	Smoking status		
Third year	94	26.8	No	247	70.4
Fourth year	49	14.0	Yes	104	29.6
Masters	49	14.0	Total	351	100.0
Total	351	100.0	Health status (self‐reported)		
Faculty			Good	235	67.0
Mathematical, physical and biological science	146	41.6	Moderate	102	29.0
Social science	80	22.8	Sick	14	4.0
Arts	67	19.1	Total	351	100.0
Business	58	16.5	Doing exercise regularly		
Total	351	100.0	No	216	61.5
Type of accommodation			Yes	135	8.5
Hall/mess	299	85.2	Total	351	100.0
Home	52	14.8	Having academic pressure		
Total	351	100.0	No	214	61.0
Family type			Yes	137	39.0
Nuclear	214	61.0	Total	351	100.0
Joint	137	39.0	Choice of course		
Total	351	100.0	Own Choice	180	51.3
Residence (urban or rural)			Force by parents	75	21.4
Rural	233	66.4	By chance	77	21.9
Urban	118	33.6	Others	19	5.4
Total	351	100.0	Total	351	100.0

From the results, it is evident that more than 60% are from rural areas and the nuclear family. In Bangladesh, the course choice depends on different factors. Sometimes students do not study their course of choice because of family choice, peer pressure, not getting admission, financial difficulties, and so on. However, it is evident that more than half of the students continue their studies by their own choice. More than 20% of students' course choice is forced by their parents and by chance. Only about 40% of students reported that they have academic pressure, including spending more time to complete assignments, lab work, being unable to understand the contents of the courses, and so on (Table 1).

The average age of the students is about 21 years with a minimum of 18 years and a maximum of 28 years. The average grade point average (GPA) in both the Secondary School Certificate (SSC) and Higher Secondary Certificate (HSC) is just above 4.5 on a scale of 5.0. In the case of SSC, the minimum GPA is 3.0, whereas the minimum GPA is 3.25 in HSC. In both cases, the maximum perfect score, that is, 5.0. However, at the university level, the average cumulative GPA (CGPA) is about 3.5 on a scale of 4.0 with a minimum of 2.61 and a maximum of 4.0. The results show that the average sleeping time of the students is just above 8 h a day. Surprisingly, some students do not spend any time on their academic studies in a week. However, the maximum study hour in a week of the students is 60 h and has an average of more than 18 h in a week. The average monthly family income of the respondents is more than 31,000 Taka (Bangladeshi currency) and the average monthly expenditure of the students is more than 8000 Taka. Only 12% of students spend more than 20,000 Taka in a month (Table 2).

**Table 2:** Summary statistics of some selected variables

Variables	Minimum	Maximum	Mean	Std. deviation	Skewness
Age (years)	18.00	28.00	21.7037	1.65718	0.574
SSC result (GPA)	3.00	5.00	4.7334	0.40574	−2.103
HSC results (GPA)	3.25	5.00	4.5977	0.40116	−0.849
B.Sc. results (CGPA)	2.61	4.00	3.4169	0.30002	−0.620
Daily hours of sleep (day + night)	3.00	14.00	8.1128	1.79309	0.395
Weekly time spent on studies (h)	0.00	60.00	18.5242	10.11442	0.341
Weekly time spent on work	0.00	72.00	10.9915	7.30107	1.638
Monthly family income (BDT)	3000.00	400,000.00	31,076.9231	30,995.98698	7.704
Your monthly expenditure (BDT)	2000.00	70,000.00	8327.0655	9838.11435	3.104

Abbreviations: BDT, Bangladeshi Taka; B.Sc., Bachelor of Science; CGPA, cumulative grade point average; GPA, grade point average; HSC, Higher Secondary Certificate; SSC, Secondary School Certificate.

**Figure 1:**
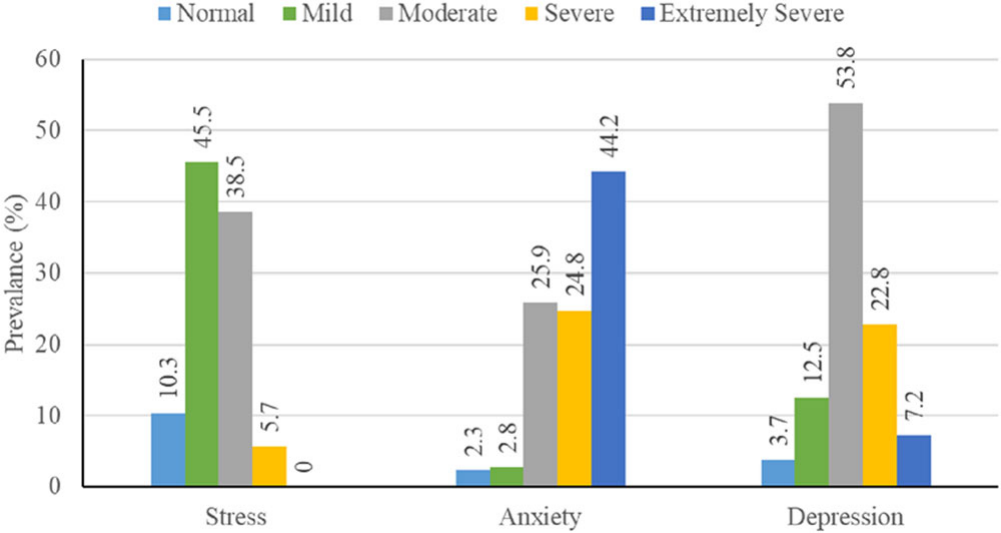
Stress, anxiety, and depression status of the students

The results presented in Figure 1 depicts the stress, anxiety, and depression level of the respondents. In each case, there are five levels such as normal, mild, moderate, severe, and extremely severe. The results illustrate that no students have extremely severe stress levels. Only about 5% of students have a severe level of stress and the majority of students have mild or moderate level stress. However, in the case of anxiety, more than 40% of students have an extremely severe level. About one out of four students have moderate or severe anxiety levels. The results suggest that just above 7% of the students have extremely severe level depression and more than half of the students have a moderate level of depression (Figure 1).

Among the variables, only two variables like gender and residence (urban or rural) are associated with the level of stress. The results indicate that the association between gender and stress level is statistically significant at a 10% level of significance. Results also depict that females have more stress than their counterparts at moderate and severe stress levels. The reason behind this is that male students are capable of absorbing more stress than female students. It is also observed that the residence (urban or rural), that is, the students are from urban or rural areas are related to the stress level. This association is statistically significant at a 5% level of significance. At the upper level of stress, students who come from rural areas are more stressed than the students who lived in urban areas (Table 3).

**Table 3:** Association of stress, anxiety, and depression status with selected variables

	Stress status							
Variable	Normal	Mild	Moderate	Severe	Extremely severe	Total	*χ* ^2^	*p* value
Gender								
Male	25 (11.7)	106 (49.5)	72 (33.6)	11 (5.1)	‐	214 (100)	6.57	0.087
Female	11 (8.0)	54 (39.4)	63 (46.0)	9 (6.6)	‐	137 (100)		
Residence (urban or rural)								
Rural	27 (11.6)	94 (40.3)	97 (41.6)	15 (6.4)	‐	233 (100)	7.85	0.049
Urban	9 (7.6)	66 (55.9)	38 (32.2)	5 (4.2)	‐	118 (100)		

	Anxiety status							
Variable	Normal	Mild	Moderate	Severe	Extremely severe	Total	*χ* ^2^	*p* value
Gender								
Male	8 (3.7)	7 (3.3)	64 (29.9)	56 (26.2)	79 (36.9)	214 (100)	15.75	0.003
Female	0 (0.0)	3 (2.2)	27 (19.7)	31 (22.6)	76 (55.5)	137 (100)		
Residence (urban or rural)								
Rural	8 (3.4)	8 (3.4)	55 (23.6)	58 (24.9)	104 (44.6)	233 (100)	6.36	0.174
Urban	0 (0.0)	2 (1.7)	36 (30.5)	29 (24.6)	51 (43.2)	118 (100)		
Family type								
Nuclear	5 (2.3)	10 (4.7)	49 (22.9)	46 (21.5)	104 (48.6)	214 (100)	13.19	0.01
Joint	3 (2.2)	0 (0.0)	42 (30.7)	41 (29.9)	51 (37.2)	137 (100)		

	Depression status							
Variable	Normal	Mild	Moderate	Severe	Extremely severe	Total	*χ* ^2^	*p* value
Gender								
Male	10 (4.7)	28 (13.1)	122 (57.0)	42 (19.6)	12 (5.6)	214 (100)	6.72	0.152
Female	3 (2.2)	16 (11.7)	67 (48.9)	38 (27.7)	13 (9.5)	137 (100)		
Type of accommodation								
Hall/mess	9 (3.0)	39 (13.0)	164 (54.8)	63 (21.1)	24 (8.0)	299 (100)	8.36	0.079
Home	4 (7.7)	5 (9.6)	25 (48.1)	17 (32.7)	1 (1.9)	52 (100)		
Family type								
Nuclear	12 (5.6)	22 (10.3)	108 (50.5)	58 (27.1)	14 (6.5)	214 (100)	13.48	0.009
Joint	1 (0.7)	22 (16.1)	81 (59.1)	22 (16.1)	11 (8.0)	137 (100)		
Birth order								
1st Child	3 (2.5)	18 (14.8)	60 (49.2)	28 (23.0)	13 (10.7)	122 (100)	30.96	0.002
2nd Child	2 (1.2)	20 (11.8)	100 (59.2)	37 (21.9)	10 (5.9)	169 (100)		
3rd Child	4 (9.5)	4 (9.5)	21 (50.0)	11 (26.2)	2 (4.8)	42 (100)		
4th Child and more	4 (22.2)	2 (11.1)	8 (44.4)	4 (22.2)	0 (0.0)	18 (100)		

In the case of the status of anxiety, there are three variables are related to them. They are gender, residence (urban or rural), and type of family. It is surprising that the selected students have either moderate or higher anxiety. More than half of the female students have extremely severe anxiety levels, whereas just above 35% of male students have extremely severe anxiety levels. Family type is also an important factor in the stress level of the students. Students who came from a nuclear family had a more extremely severe level of anxiety compared to the students from joint families (Table 3).

The results of the depression level and other variables are presented in Table 3. The findings reveal that the type of accommodation of the students, their family type, and birth order are associated with depression at a 5% level of significance. Normally the students who lived in a hall or mess have more depression compared to the students who live with their family members at home. The results suggest that the first child of the parents has a higher level of depression compared to the other child of the parents. In Bangladesh, generally, the first child is more responsible than another child of the parents (Table 3).

The *χ* ^2^ and normed *χ* ^2^ $\left(\chi^{2}/d\right)$ are used to check the lack of fit. The result depicts that our model is good fitted. The RMSEA is the second fit statistic, and in a well‐fitting model, the lower limit should be close to 0, while the upper limit should be less than 0.08. The RMR is the square root of the difference between the residuals of the sample covariance matrix and the hypothesized covariance model. Values for the NFI range between 0 and 1 and it is recommended that values greater than 0.90 indicate a good fit. Also, the value of CFI lies between 0.0 and 1.0, with values closer to 1.0 indicating a good fit. Moreover, adjusted goodness‐of‐fit statistic (AGFI), which adjusts the GFI based on degrees of freedom and values for the AGFI, like the GFI, range from 0 to 1, and values of 0.90 or higher are generally considered to reflect well‐fitting models. For this study, the quality of fit measurements of the chosen model stated that the fit of the model was satisfactory (Table 4).

**Table 4:** Goodness‐of‐fit indices of confirmatory factor analysis

Model fit index	Good fit	Acceptable fit	Model values
*χ* ^2^ (df)	Nonsignificant	Nonsignificant	418.938 (186), *p* < 0.001
Normed χ^2^ $\left(\chi^{2}/d\right)$	$\left(\chi^{2}/d\right)<3$	$3<\left(\chi^{2}/d\right)<5$	2.252
RMSEA	0 < RMSEA < 0.05	0.05 < RMSEA < 0.08	0.06
RMR	0 ≤ RMR ≤ 0.05	0.05 < RMR < 0.1	0.057
NFI	0.97 ≤ NFI ≤ 1	0.95 ≤ NFI ≤ 0.97	0.952
CFI	0.97 ≤ CFI ≤ 1	0.95 ≤ CFI ≤ 0.97	0.945
GFI	0.95 ≤ GFI ≤ 1	0.90 ≤ GFI ≤ 0.95	0.918
AGFI	0.90 ≤ AGFI ≤ 1	0.85 ≤ AGFI ≤ 0.90	0.865

Abbreviations: AGFI, adjusted goodness‐of‐fit index; CFI, comparative fit index; GFI, goodness‐of‐fit index; NFI, normed‐fit index; RMR, root mean square residual; RMSEA, root mean square error of approximation.

**Figure 2:**
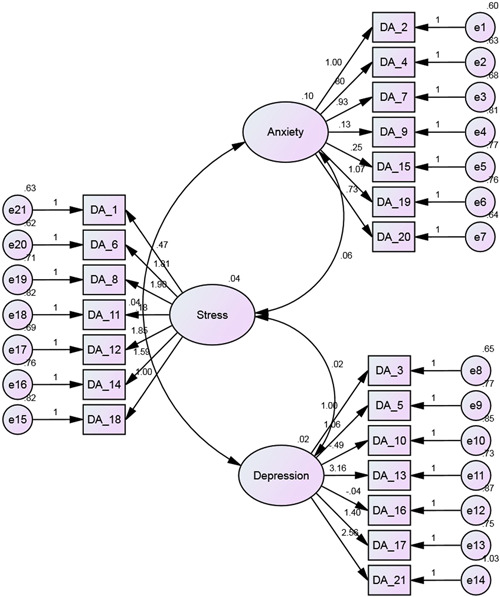
The results of the Depression, Anxiety and Stress Scale‐21 Items model with path coefficients

A CFA was carried out here to address the structure of the DASS‐21 instrument factors. The structural equation model (SEM) was also used to validate the DASS‐21 instrument factor structure. This approach is used for the study and elaboration of the interrelationships between DASS items in accordance with their usual underlying factors. The results with path coefficients of the SEM model are presented in Figure 2. The path coefficients of the items for measuring stress lie between 0.04 and 1.98 and indicate that some of the items influence the stress level approximately 1.98 times. The results also depict that the path coefficients of anxiety items vary from 0.13 to 1.07, and for depression, the highest value of the coefficients is 3.16. The findings also reveal that the three components of measuring mental health are interconnected (Figure 2).

## 4 DISCUSSION

This study aims to identify the level of depression, anxiety, and stress among students and find out its relationship to the socioeconomic status of the students. Findings reveal that approximately 45% of the respondents have moderate to severe levels of stress; however, no student has an extreme level of stress. About 50% of students have moderate to severe anxiety, and shockingly, more than 44% of students have an extremely severe level of anxiety. Moreover, more than half of the students have moderate depression levels and about 22% and 7% of students have severe and extremely severe levels of depression, respectively. In this study, the rates of depression and anxiety among students were lower than those seen in the recent COVID‐19 pandemic.^42^, ^47^, ^48^ However, before COVID‐19, a survey revealed that 6.2% of students suffered from severe depression,^49^ which is consistent with our study findings.

Several sociodemographic characteristics, especially age, gender, examination criteria, dissatisfaction, and interminable test schedules, have been recognized as contributing factors to stress, anxiety, and depression.^50^, ^51^ Previous studies also pointed out that higher levels of depression or stress were linked to demographic characteristics such as age and sex,^42^ which supports our study results. Gender, on the other hand, has shown mixed outcomes. For example, researchers discovered that female students were more stressed than male students,^52^ but another study revealed no significant gender differences in stress.^47^ Furthermore, a variety of academic and psychosocial challenges, such as high parental expectations, the breadth of the academic curriculum, sleeping issues, future worries, loneliness, and living in university dorms, were major causes of anxiety and depression among students.^53^, ^54^, ^55^ Our study is supported by some of these findings. Students who awoke from sleep multiple times had more mental health issues than others.^56^

Female students have more stress than their counterparts at the moderate and severe level and our findings are supported by another study.^57^ The reason behind that male students are capable of absorbing more stress than female students.^58^ Men may also be more likely to believe in their own ability to control the situation, which is a protective factor against anxiety disorders.^57^ It is also observed that the residence (urban or rural), that is, the students are from urban or rural areas are related to the stress level. At the upper level of stress, students who came from rural areas are more stressed than the students who lived in urban areas. The reason behind this may be the lack of facilities, financial vulnerabilities, poor living conditions, and so on.^59^ Another study highlighted that students who live in cities are more stressed than those who reside in rural areas.^56^ However, a study pointed out that there is no significant difference to build symptoms of depression, anxiety, and stress among urban and rural students.^58^ Students who come from a nuclear family have a higher level of anxiety than students who come from a joint family. Our study findings differ from other studies where the authors mentioned that depression, anxiety, and stress symptoms are similar across students in nuclear and joint family systems.^58^, ^60^

The findings reveal that the type of accommodation of the students and birth order are associated with depression. Normally the students who lived in a hall or mess have more depression compared to the students who live with their family members at home. But other studies highlighted that students who live with family have more depression and stress symptoms than those who live with friends or alone, while anxiety levels were similar in both groups.^61^, ^62^, ^63^ The results suggest that the first child of the parents has a higher level of depression compared to the other child of the parents. In Bangladesh, traditionally it is believed that the first child is more responsible than another child of the parents. Furthermore, the results with path coefficients of the SEM model depict that the scores in the three domains (depression, anxiety, and stress) were found to be correlated and these findings are supported by other previous studies.^58^, ^60^, ^64^

## 5 LIMITATIONS OF THE STUDY

This study has some limitations. First, it has been carried out with self‐funding; therefore, the authors were not able to collect a large amount of data. Second, this is a cross‐sectional study, as a result, causal inference is not possible. Third, the small sample is collected from one university of Bangladesh; hence, it may not be generalizable to the population from the whole country.

## 6 CONCLUSION

The study findings indicate that university students have some sort of depression, anxiety, and stress. Sex, residence (urban or rural), birth order, type of accommodation, and family type of the students acted as risk factors for depression, stress, and anxiety among students. The CFA indicates that there exist interrelationships between DASS items in accordance with their common underlying factors. Given the harmful impacts of stress on academic performance and health, university administrators should incorporate anxiety, stress, and depression management training in orientation activities. A better method may be the use of a workshop related to stress, anxiety, and depression management. Certainly, anxiety, stress, and depression in the university setting cannot be eliminated, but it would be possible to do a better job preparing students to manage it. This study considers only one university; therefore, the results may not represent the overall country's situation. Further study may include more samples and more universities to generalize the results.

## CONFLICT OF INTEREST

The authors declare no conflicts of interest.

## ETHICS STATEMENT

This study was conducted while maintaining ethical standards to the highest possible extent. All participants read, understood, and provided their consent for publishing the analyzed results of this survey without their identifiable information.

## AUTHOR CONTRIBUTIONS

**Md. Moyazzem Hossain**: Conceptualization; data curation; formal analysis; methodology; software; supervision; visualization; writing—original draft; writing—review and editing. **Md. Asraful Alam**: Conceptualization; data curation; writing—original draft. **Monirul Hasan Masum**: Conceptualization; formal analysis; methodology; software; visualization; writing—original draft. All authors have read and approved the final version of the manuscript. All authors take complete responsibility for the integrity of the data and accuracy of the data.

## Data Availability

The data that support the findings of this study are available from the corresponding author upon reasonable request.

## References

[1] The World Health Report—reducing risks, promoting healthy life . World Health Organization. 2002. Accessed April 11, 2021. https://www.who.int/whr/2002/en/

[2] Mental health action plan—2020 . World Health Organization. 2013. Accessed April 11, 2021. https://www.who.int/publications/i/item/9789241506021

[3] Seligman MEP , Walker EF , Rosenhan DL . Abnormal Psychology. 4th ed. W. W. Norton & Company; 2001:1‐864. https://www.amazon.co.uk/Abnormal-Psychology-Martin-P-Seligman/dp/039394459X/ref=sr_1_1?dchild=1%26hvadid=80676698098422%26hvbmt=be%26hvdev=c%26hvqmt=e%26;keywords=abnormal%2Bpsychology%2Bseligman%26qid=1617383233%26sr=8-1

[4] American Psychiatric Association. Diagnostic and Statistical Manual of Mental Disorders (DSM‐5®). *American Psychiatric Publishing*. May 22, 2013. Accessed June 18, 2020.

[5] Pharmacological treatment of mental disorders in primary health care . World Health Organization. 2009. Accessed April 11, 2021. https://www.who.int/mental_health/management/psychotropic/en/ 23762966

[6] Testa A , Giannuzzi R , Sollazzo F , Petrongolo L , Bernardini L , Daini S . Psychiatric emergencies (part I): psychiatric disorders causing organic symptoms. Eur Rev Med Pharmacol Sci. 2013;17(suppl 1):55‐64. 23436668

[7] Testa A , Giannuzzi R , Sollazzo F , Petrongolo L , Bernardini L , Dain S . Psychiatric emergencies (part II): psychiatric disorders coexisting with organic diseases. Eur Rev Med Pharmacol Sci. 2013;17(suppl 1):65‐85. 23436669

[8] Testa A , Giannuzzi R , Daini S , Bernardini L , Petrongolo L , Gentiloni Silveri N . Psychiatric emergencies (part III): psychiatric symptoms resulting from organic diseases. Eur Rev Med Pharmacol Sci. 2013;17(suppl 1):86‐99. 23436670

[9] Smoller JW , Andreassen OA , Edenberg HJ , Faraone SV , Glatt SJ , Kendler KS . Psychiatric genetics and the structure of psychopathology. Mol Psychiatry. 2019;24(3):409‐420. 10.1038/s41380-017-0010-4 29317742 PMC6684352

[10] Craske MG , Stein MB , Eley TC , et al. Anxiety disorders. Nat Rev Dis Prim. 2017;3(1):17024. 28470168 10.1038/nrdp.2017.24 PMC11009418

[11] Kendler KS . Major depression and generalised anxiety disorder. Focus. 2004;2(3):416‐425.

[12] Kessler RC , Berglund P , Demler O , et al. The epidemiology of major depressive disorder: results from the National Comorbidity Survey Replication (NCS‐R). J Am Med Assoc. 2003;289(23):3095‐3105. 10.1001/jama.289.23.3095 12813115

[13] Khanam SJ , Bukhari SR . Depression as a predictor of academic performance in male and female university students. J Pakistan Psychiatr Soc. 2015;12(2):1‐15.

[14] Kaur S , Deepti SS , Lal M . Prevalence and correlates of depression among college going students of District Amritsar, India. Int Res J Med Sci. 2014;2(11):5‐9.

[15] Alvi T , Assad F , Ramzan M , Khan FA . Depression, anxiety and their associated factors among medical students. J Coll Physicians Surg Pakistan. 2010;20(2):122‐126. 20378041

[16] Kaya M , Genç M , Kaya B , Pehlivan E . Prevalence of depressive symptoms, ways of coping, and related factors among medical school and health services higher education students. Turk Psikiyatr Derg. 2007;18(2):137‐146. 17566879

[17] Adewuya AO , Ola BA , Afolabi OO . Validity of the Patient Health Questionnaire (PHQ‐9) as a screening tool for depression amongst Nigerian university students. J Affect Disord. 2006;96(1–2):89‐93. 16857265 10.1016/j.jad.2006.05.021

[18] Daniel K . Loneliness and depression among university students in Kenya. Glob J Hum Soc Sci Arts Humanit. 2013;13(4):11‐18.

[19] Kumaraswamy I . Academic stress, anxiety and depression among college students—a brief review. Int Rev Soc Sci Humanit. 2013;5(1):135‐143.

[20] Uehara T , Takeuchi K , Kubota F , Oshima K , Ishikawa O . Annual transition of major depressive episode in university students using a structured self‐rating questionnaire. Asia‐Pacific Psychiatry. 2010;2(2):99‐104.

[21] Andrews B , Wilding JM . The relation of depression and anxiety to life‐stress and achievement in students. Br J Psychol. 2004;95(4):509‐521. 15527535 10.1348/0007126042369802

[22] Selye H . The Stress of Life. McGraw‐Hill; 1956.

[23] Lazarus RS , Folkman S . Stress, Appraisal, and Coping. Springer Publishing; 1984:1‐60.

[24] Lai PC , Chao WC , Chanf YY , Chang TT . Adolescent Psychology. National Open University; 1996.

[25] Vijaya VS , Karunakaran P . A study on stress among intermediate students. Innov Thoughts Int Res J. 2013;1:21‐25.

[26] Asif S , Mudassar A , Shahzad TZ , Raouf M , Pervaiz T . Frequency of depression, anxiety and stress among university students. Pakistan J Med Sci. 2020;36(5):971‐976. 10.12669/pjms.36.5.1873 PMC7372668 32704273

[27] Herrmann K , Déchelotte P , Ladner J , Tavolacci MP . Depression, anxiety stress and associated factors among university students in France. Eur J Public Health. 2019;29(suppl 4):ckz186.555.

[28] Cheung K , Tam KY , Tsang H , Zhang LW , Lit SW . Depression, anxiety and stress in different subgroups of first‐year university students from 4‐year cohort data. J Affect Disord. 2020;274:305‐314. 32469820 10.1016/j.jad.2020.05.041

[29] Teh C , Ngo C , Zulkifli R , Vellasamy R , Suresh K . Depression, anxiety and stress among undergraduate students: a cross sectional study. Open J Epidemiol. 2010;5:260‐268. 10.4236/ojepi.2015.54030

[30] Wahed YAW , Hassan SK . Prevalence and associated factors of stress, anxiety and depression among medical Fayoum University students. Alexandria J Med. 2017;53(1):77‐84.

[31] Islam S , Akter R , Sikder T , Griffiths MD . Prevalence and factors associated with depression and anxiety among first‐year university students in Bangladesh: a cross‐sectional study. Int J Ment Health Addict. 2020:1‐14. 10.1007/s11469-020-00242-y

[32] Liu F , Zhou N , Cao H , et al. Chinese college freshmen's mental health problems and their subsequent help‐seeking behaviors: a cohort design (2005–2011). PLOS ONE. 2017;12(10):e0185531. 29040266 10.1371/journal.pone.0185531 PMC5644985

[33] Pereira S, Early N, Outar L, Dimitrova M, Walker L, Dzikiti C. *University Student Mental Health Survey*. 2020. Accessed May 26, 2021. https://www.diginbox.com

[34] Beiter R , Nash R , McCrady M , et al. The prevalence and correlates of depression, anxiety, and stress in a sample of college students. J Affect Disord. 2015;173:90‐96. 25462401 10.1016/j.jad.2014.10.054

[35] Bruffaerts R , Mortier P , Kiekens G , et al. Mental health problems in college freshmen: prevalence and academic functioning. J Affect Disord. 2018;225:97‐103. 28802728 10.1016/j.jad.2017.07.044 PMC5846318

[36] Mehr KE , Daltry R . Examining mental health differences between transfer and nontransfer university students seeking counseling services. J College Stud Psychother. 2016;30(2):146‐155.

[37] Cuttilan AN , Sayampanathan AA , Ho RCM . Mental health issues amongst medical students in Asia: a systematic review [2000–2015]. Ann Transl Med. 2016;4(4):72. 27004219 10.3978/j.issn.2305-5839.2016.02.07 PMC4779785

[38] Tung YJ , Lo KKH , Ho RCM , Tam WSW . Prevalence of depression among nursing students: a systematic review and meta‐analysis. Nurse Educ Today. 2018;63:119‐129. 29432998 10.1016/j.nedt.2018.01.009

[39] Hamaideh SH , Al‐Modallal H , Tanash M . Hamdan‐Mansour A. Depression, anxiety and stress among undergraduate students during COVID‐19 outbreak and “home‐quarantine”. Nurs Open. 2021;9:1‐9. 10.1002/nop2.918 PMC8242644 33988913

[40] Hosen I , Al Mamun F , Mamun MA . The role of sociodemographics, behavioral factors, and internet use behaviors in students' psychological health amid COVID‐19 pandemic in Bangladesh. Health Sci Rep. 2021;4(4):e398. 34622029 10.1002/hsr2.398 PMC8485611

[41] Lee J , Jeong HJ , Kim S . Stress, Anxiety, and Depression Among Undergraduate Students during the COVID‐19 Pandemic and their Use of Mental Health Services. Innov High Educ. 2021;46(5):519‐538. 10.1007/s10755-021-09552-y PMC8062254 33907351

[42] Karing C . Prevalence and predictors of anxiety, depression and stress among university students during the period of the first lockdown in Germany. J Affect Disord Rep. 2021;5:100174. 34642682 10.1016/j.jadr.2021.100174 PMC8497174

[43] Wang C , Wen W , Zhang H , et al. Anxiety, depression, and stress prevalence among college students during the COVID‐19 pandemic: a systematic review and meta‐analysis. J Am Coll Health. 2021:1‐8. 10.1080/07448481.2021.1960849 34469261

[44] Yeasmin S , Banik R , Hossain S , et al. Impact of COVID‐19 pandemic on the mental health of children in Bangladesh: a cross‐sectional study. Child Youth Serv Rev. 2020;117:105277. 32834275 10.1016/j.childyouth.2020.105277 PMC7387938

[45] Lovibond PF , Lovibond SH . The structure of negative emotional states: comparison of the Depression Anxiety Stress Scales (DASS) with the Beck Depression and Anxiety Inventories. Behav Res Ther. 1995;33(3):335‐343. 7726811 10.1016/0005-7967(94)00075-u

[46] Cronbach LJ . Coefficient alpha and the internal structure of tests. Psychometrika. 1951;16(3):297‐334.

[47] Fu W , Yan S , Zong Q , et al. Mental health of college students during the COVID‐19 epidemic in China. J Affect Disord. 2021;280(pt A):7‐10. 10.1016/j.jad.2020.11.032 PMC7656159 33197782

[48] Wang X , Hegde S , Son C , Keller B , Smith A , Sasangohar F . Investigating mental health of US college students during the COVID‐19 pandemic: cross‐sectional survey study. J Med Internet Res. 2020;22(9):e22817. 32897868 10.2196/22817 PMC7505693

[49] Choi B , Shim G , Jeong B , Jo S . Data‐driven analysis using multiple self‐report questionnaires to identify college students at high risk of depressive disorder. Sci Rep. 2020;10(1):1‐13. 32398788 10.1038/s41598-020-64709-7 PMC7217968

[50] Alvi T , Assad F , Ramzan M , Khan FA . Depression and anxiety among medical students. J Coll Physicians Surg Pakistan. 2010;20(2):122‐126. 20378041

[51] Mirza AA , Baig M , Beyari GM , Halawani MA , Mirza AA . Depression and anxiety among medical students: a brief overview. Adv Med Educ Pract. 2021;12:393‐398. 33911913 10.2147/AMEP.S302897 PMC8071692

[52] Elmer T , Mepham K , Stadtfeld C . Students under lockdown: comparisons of students' social networks and mental health before and during the COVID‐19 crisis in Switzerland. PLOS ONE. 2020;15(7):e0236337. 32702065 10.1371/journal.pone.0236337 PMC7377438

[53] Rab F , Mamdou R , Nasir S . Rates of depression and anxiety among female medical students in Pakistan. East Mediterr Health J. 2008;14(1):126‐133. 18557460

[54] Saeed H , Saleem Z , Ashraf M , et al. Determinants of anxiety and depression among university students of Lahore. Int J Ment Health Addict. 2018;16(5):1283‐1298.

[55] Hossain MM , Rahman MH . Assessing sleep quality and its effects on academic performance among university students. J Sleep Sci. 2020;5(2):67–72.

[56] Alam MK , Ali FBin , Banik R , Yasmin S , Salma N . Assessing the mental health condition of home‐confined university level students of Bangladesh due to the COVID‐19 pandemic. J Public Health. 2021:1‐8. 10.1007/s10389-021-01542-w PMC8053029 33898164

[57] Hosseini L , Khazali H . Comparing the level of anxiety in male & female school students. Procedia Soc Behav Sci. 2013;84:41‐46.

[58] Haq MAul , Dar IS , Aslam M , Mahmood QK . Psychometric study of depression, anxiety and stress among university students. J Public Health. 2017;26(2):211‐217.

[59] Christie H , Munro M , Rettig H . Accommodating students. J Youth Stud. 2002;5(2):209‐235.

[60] Bhasin SK , Sharma R , Saini NK . Depression, anxiety and stress among adolescent students belonging to affluent families: a school‐based study. Indian J Pediatr. 2010;77(2):161‐165. 19936655 10.1007/s12098-009-0260-5

[61] Lester D . Depression and suicidal ideation in college students: a preliminary study of campus variables. Psychol Rep. 2013;112(1):106‐108. 23654031 10.2466/12.02.10.PR0.112.1.106-108

[62] Shamsuddin K , Fadzil F , Ismail WSW , et al. Correlates of depression, anxiety and stress among Malaysian university students. Asian J Psychiatr. 2013;6(4):318‐323. 23810140 10.1016/j.ajp.2013.01.014

[63] Abdallah AR , Gabr HM . Depression, anxiety and stress among first year medical students in an Egyptian public university. Int Res J Med Med Sci. 2014;2(1):11‐19.

[64] Riaz A , Kamal S , Butt NS . Psychometric analysis of depression, anxiety and stress among women of Wazirabad City. Casp J Appl Sci Res. 2013;2(10):61‐68.

